# When Should We Use Care Robots? The Nature-of-Activities Approach

**DOI:** 10.1007/s11948-015-9715-4

**Published:** 2015-11-07

**Authors:** Filippo Santoni de Sio, Aimee van Wynsberghe

**Affiliations:** 1 Section of Ethics/Philosophy of Technology, Faculty of Technology, Policy and Management, Delft University of Technology, Jaffalaan 5, 2628 BX Delft, The Netherlands; 2 Department of Philosophy, University of Twente, Postbox 217, 7500 AE Enschede, The Netherlands

**Keywords:** Care robot ethics, Robot ethics, Nature-of-activities approach, Care Centered Value Sensitive Design, Value Sensitive Design

## Abstract

When should we use care robots? In this paper we endorse the shift from a simple normative approach to care robots ethics to a complex one: we think that one main task of a care robot ethics is that of analysing the different ways in which different care robots may affect the different values at stake in different care practices. We start filling a gap in the literature by showing how the philosophical analysis of the nature of healthcare activities can contribute to (care) robot ethics. We rely on the nature-of-activities approach recently proposed in the debate on human enhancement, and we apply it to the ethics of care robots. The nature-of-activities approach will help us to understand why certain practice-oriented activities in healthcare should arguably be left to humans, but certain (predominantly) goal-directed activities in healthcare can be fulfilled (sometimes even more ethically) with the assistance of a robot. In relation to the latter, we aim to show that even though all healthcare activities can be considered as practice-oriented, when we understand the activity in terms of different legitimate ‘fine-grained’ descriptions, the same activities or at least certain components of them can be seen as clearly goal-directed. Insofar as it allows us to ethically assess specific functionalities of specific robots to be deployed in well-defined circumstances, we hold the nature-of-activities approach to be particularly helpful also from a design perspective, i.e. to realize the Value Sensitive Design approach.

## Introduction

A revolution in healthcare is before us, one that involves the introduction of robots both in the surgical suite and throughout the rest of the institution. Ethical reasons for and against this robot revolution in healthcare have been presented in recent years (Lin et al. 2011). On the one hand, it has been argued that robots hold the promise to mitigate the shortage of healthcare workers and resources (Veruggio and Operto 2008), and that they can enhance the autonomy of elderly persons (Sorell and Draper 2014). On the other hand, the concern has been raised that robots in healthcare will displace workers and will change the moral quality and standard of care for the worse (Sparrow and Sparrow 2006; Decker 2008; Coeckelbergh 2010; Vallor 2011; Sharkey 2014). Added to these concerns, other scholars insist that the ethical issues of robots in general are of the utmost concern, for example, responsibility (Dante and Tamburrini 2006), privacy (Calo 2011) and/or agency (Sullins 2006; Wallach and Allen 2010). Despite the multiple perspectives on the most pressing ethical issues to address, the necessity of a more nuanced approach has also been recently stressed (Asaro 2006; van Wynsberghe 2015).

What all scholars can agree on is that there seems to be no simple answer to the question of whether or not the introduction of robots will change healthcare practices for the better or for the worse. In order make way for a well-balanced answer to this question a systematic reflection on the different values that are at stake in healthcare practices has been invoked together with an analysis of the way in which these values may be fostered or endangered by the introduction of (various) robots (Vallor 2011, Sharkey and Sharkey 2012). Finally, also the necessity of a shift from the ethical analysis of the *use* to the definition of the ethical requirements for the *design* of care robots has been stressed (van Wynsberghe 2012, 2013, 2015).

We endorse the shift from a simple normative approach to a complex one: we think that the main task of a care robot ethics is, at least at this early stage, that of analyzing the different ways in which different care robots may affect the different values at stake in different care practices; not just analyzing the impact of care robots on the basis of one prominent ethical consideration or approach (e.g. autonomy or one particular conception of human dignity). We also endorse the Value Sensitive Design turn in care robot ethics proposed by van Wynsberghe as we agree that normative considerations have to be embedded in the *design process* of care robots since the early stages of it; they should not come into play only in the evaluation of the technology (i.e. retrospectively).

In this paper we start filling a gap in the literature on care robots by showing how one particular philosophical framework, i.e. the analysis of *the nature of healthcare activities*, can contribute to the twofold goal of a normative complex and design-oriented care robot ethics. As it were, we aim to add some tools from the *philosophy of action* to the toolbox of the care robot ethicist. In order to do so we rely on the *nature*-*of*-*activities* approach recently proposed by Santoni de Sio et al. (2014, in press) in the debate on human enhancement. Based on this approach we come to the following conclusions; (1) the permissibility or impermissibility of the use of care robots is a normative decision to be taken by balancing different value considerations at stake in different healthcare practices; (2) ethical analysis can be significantly enriched by attending to certain conceptual distinctions about the nature of healthcare activities.

In particular, the nature-of-activities approach will help us to understand why certain *practice*-*oriented* activities in healthcare should arguably be left to humans, but certain (predominantly) *goal*-*directed* activities in healthcare can be fulfilled (sometimes even more ethically) with the assistance of a robot. In relation to the latter, we aim to show that even though all healthcare activities can be considered as practice-oriented, when we understand the activity in terms of different legitimate ‘fine-grained’ descriptions, the same activities or at least certain components of them can be seen as clearly goal-directed. In this way, we also give a more precise content to the traditional idea that both means and ends are relevant for the ethical evaluation of an action.

One general methodological conclusion we will reach is that in order to make sense of the many different, sometimes contrasting values present in care activities, we may need to refer at the same time to different and sometime distant philosophical traditions: virtue ethics and care ethics, the capability approach, the liberal tradition. While aware of the important differences between these traditions, with our approach based on the conceptual analysis of action, we aim to make sense of the respective relevance for care activities of (care) values highlighted in these different traditions, i.e. the realization of carers skill and a meaningful relationship between carer and patient (Vallor 2011), the patient’s dignity (Sharkey 2014) but also the patient’s moral autonomy (Sorell and Draper 2014); we also want to explore the possibility of some reconciliation between these values under some circumstances. This reference to different philosophical traditions with their own specific language requires us to establish some clear terminological convention to avoid confusion. In the care ethics tradition, the label of “care practice” is used; however, given our use of the expression “practice-oriented activities” we will refer to care practices as “care activities”: to avoid confusion, and to leave the possibility open to include in our ethical analysis of care activities also values traditionally associated with traditions other than care ethics.

One substantive ethical claim we will make is that it is *permissible* to delegate (at least) the goal-directed portion of a care activity to a care robot, provided such a delegation is done prudentially to ensure that the robot is not delegated a role or responsibility that coincides with the practice-oriented portion of the care practice.

Finally, we hold the nature-of-activities approach to improve the Value Sensitive Design approach (VSD) (Friedman et al. 2003; van den Hoven 2007) and, more particularly, the Care-Centered Value Sensitive Design approach (CCVSD) (van Wynsberghe 2012, 2013, 2015), according to which (care) values should be embedded in the design of (care) technologies. In fact, following the idea that VSD cannot be realized if we don’t have a clear methodology to establish *which* values we want to promote (Manders-Huits 2011), in this paper we propose a new methodology to: (a) identify a broader platform of values potentially relevant in care activities; (b) establish which values should be embedded in specific functionalities of specific robots to be deployed in parts or portions of different care activities under well-defined circumstances. This will arguably put us in a better position to: (c) recognize possible tensions between values under different circumstances and to suggest ways to solve or loosen some of these tensions *by design*.

In the rest of the paper, we will: (1) give a general definition of care robots, (2) present the nature-of-activities approach, (3) apply the nature-of-activities approach to the ethics of using care robots with reference to two particular examples of care activities (lifting and urine sample collection), (4) present the design relevance of this approach through the analysis of one particular example of a care robot (the “wee-bot”), (5) draw some conclusions and set the stage for future research.

## What is a Care Robot?

There is no universal agreement on what a care robot may be defined as. We use here the idea that a care robot is a technological device integrated into care practices to assist healthcare personnel in their role as care taker (Vallor 2011; Sharkey and Sharkey 2011; van Wynsberghe 2012, 2013). The care robot may be an assistant to the surgeon as in the daVinci^®^ surgical robot or may work as an assistant to the nurse as in the TUG© robot used for delivery of sheets and medications in a hospital. Alternatively, a care robot may work directly with patients as in rehabilitative robots for stroke survivors (Lo et al. 2010) or robots used to remind patients to take their medication (Anderson and Anderson 2007). Care robots may be intended to assist in physical labour like the robots mentioned above or may be intended to be used for more social goals, like companionship, as in the Paro robot.

There is no capability or appearance standard to all care robots. They may be stationary or mobile and they may be humanoid, machine-like or creature-like in appearance. What they all have in common is their integration into the healthcare domain and, more importantly, their integration into a therapeutic relationship (whether this integration be the replacement of a traditional therapeutic relationship or an enhancement of a current one).

## The Nature-of-Activities Approach

When should we use care robots? In asking this question we are in the first place asking for the normative features and considerations that warrant the *use* of robots in healthcare.^1^ The robot will be integrated into care activities and will accomplish some kind of action within this activity; therefore, in order to assess whether the change in the activity introduced by the use of the robot is a change for better or for worse we arguably ought to explore the *nature* of those activities in the first place. In fact, in order to decide whether the introduction of robots in care activities is desirable or not, in addition to considerations such as cost-effectiveness and health benefits, we must also understand what the values involved in those activities are; and one way to identify these value is arguably to understand what the point(s) of care activities are; with this information we will then be in the position to assess if these points may be fostered, or alternatively endangered, by the introduction of care robots.

A similar approach has been put forward by Santoni de Sio et al. (2014) in the context of the ethical debate on performance-enhancing technologies. Santoni de Sio et al. (2014) have elaborated what they call a “nature-of-activities approach”, that is meant to work as a reasoning template to decide the permissibility/impermissibility/obligatoriness of the use of enhancing technologies in different contexts. The nature-of-activities approach relies on one main distinction between goal-directed and practice-oriented activities.^2^ Goal-directed activities are those in which the main point is a state of affairs that is *external* to the activity. In contrast, practice-oriented activities are those in which the main point is, or heavily depends, on the performance of the activity itself, i.e. the goal is *internal* to the activity.

Typical examples of goal-directed activities would be surgical practices and daily non-professional car driving. Here the main point (and thus “the nature”) of the activities is restoring a patient to health and reaching a certain place, respectively. As far as these goals are attained the point of the activity is satisfied.

Typical examples of practice-oriented activities are sports, intellectual activities like reading fiction, or social activities like hanging out with friends. Here the main point is the realization itself of a given performance or a series of behaviours or actions. For example, running on a certain track (as opposed to simply getting to a place), reading certain pages (as opposed to simply learning a story) or enjoying some relaxation and drinks while chatting with friends (as opposed to a simple exchange of information with them and quenching one’s thirst from time to time).

It must be recognized that most activities have both external and internal points—for instance, many professions may be seen as both goal-directed and practice-oriented, as they are aimed both at making one’s living/producing certain results *and* at practicing a certain kind of activity. However, we hold that the distinction is still helpful because: (a) in some cases (like the above-mentioned examples) it is indeed possible to identify one prominent goal of a given activity, and (b) even when there is not only one uncontroversially prominent goal, it may still be helpful to explicitly recognize what the different points of an activity are, in order to make sense of the various dimensions of that activity, and, relatedly, of the different values involved in those activities.

An additional conceptual tool to make sense of the nature of the activities in which care robots may be deployed is the analysis of activities in terms of different action-descriptions. Not only do different activities have different natures (and value) according to their different points (for instance, a chat with a friend has a different nature than a job interview), but one particular activity is also subject to different descriptions according to the kind and amount of elements that are taken into account (the deployed tools, the intentions of the agent, the institutional context, their broader effects, etc.). According to a famous example by Anscombe (1957) a man’s behaviour consisting in moving an arm up and down while holding a handle may be legitimately described, according to different circumstances or perspectives, as “pumping water”, “contracting these muscles”, “tapping out this rhythm”, “doing his job”, etc. Moreover, the same activity under the same description can also be split into different portions. A given job is made by different tasks, and all tasks can be analysed in particular subtasks.

Santoni de Sio et al. (2014) make the claim that the possibility of different descriptions of a given activity may be relevant for understanding the impact of performance-enhancing technologies on the nature of activities, and therefore also to decide the permissibility of the use of those technologies in various activities. For instance, in relation to sports activities they propose a distinction between coarse-grained and fine-grained descriptions of actions. Coarse-grained descriptions take into account only some macroscopic features of the sport activity, whereas fine-grained descriptions take into account a larger number of features. According to a coarse-grained description, a courtyard basketball game between children is as much a basketball game as an NBA Final. However, according to a fine-grained description of the activity, one that takes into account the “marked differences in rules, skill level and training present in NBA games … compared to typical courtyard basketball games, it is natural to infer that these are *two different games*” (p. 185).

The nature-of-activities approach helps make sense of what may be *wrong* with the use of performance-enhancing technologies in sport.^3^ Given that: (a) sport is a prominently practice-oriented activity, and that (b) sufficiently fine-grained descriptions of various sport activities may reveal different internal points pursued by those activities; then, *if* we have reasons to value these points, and the introduction of a certain technology will restrict the pursuit of one or more of these points, then we will have a *prima facie* argument against the introduction of that technology in that activity.

However, Santoni de Sio et al. (2014) have also suggested that the nature-of-activities approach may explain why performance-enhancing may sometimes be morally *good*. Typically, in prominently goal-directed activities, ones in which the pursuit of an external goal mainly defines the point of the activity (e.g. healing patients in a surgical operation), performance-enhancing technologies may be seen as enhancing not endangering the nature and value of the activity. As a general methodological conclusion, Santoni de Sio et al. (in press) propose that we “think reflectively and reason about what point different activities might have, and whether that point would be foregone by allowing enhancers. Each fine-grainely-defined activity may have its own specific point, this point may have a larger or smaller moral or societal value, and this value may be fostered or jeopardised by enhancers”.

The nature-of-activities approach proves to be a promising starting point for offering analytic answers to moral questions about enhancement. We argue here that this approach may be fruitfully applied also to answer moral questions about the use of care robots when viewed as enhancements to the work of nurses, physicians, other care professionals; or, possibly, as enhancing the capacities of patients.

## Care Activities and Care Robots

How might the nature-of-activities approach apply to care activities in which care robots may be deployed? Firstly, we must reflect on the nature of care activities. According to the care ethicist Joan Tronto: “the notion of a care practice is complex; it is an alternative to conceiving of care as a principle or as an emotion. To call care a practice implies that it involves both thoughts and action, that thought and action are interrelated, and that they are directed *toward some end* (Tronto 1993, p. 108, emphasis added).

With this description Tronto leaves room to interpret a care activity (i.e. a care practice)^4^ as goal-directed given that the activity is aimed at a distinct end. Consider, for example, the surgeon whose goal is to carry-out a surgical intervention or the anesthesiologist whose goal is to provide the right medications in the precise dosage throughout an operation.

Many care ethicists, however, have insisted that care activities conform mostly to the practice-oriented description provided earlier in this paper. Vallor (2011) describes care activities as a platform for the development of necessary care skills as well as skills for becoming an empathic human being. Van Wynsberghe (2012) describes care activities as the vehicle for the realization of care values. For the latter, values like human dignity and privacy are made real when the curtain is enclosed around the patient prior to bathing them in bed. Accordingly, care activities, or at least portions of care activities, are about much more than the external ends to which they aim; care activities, or at least portions of care activities, also have a practice-oriented nature.

Following Tronto, van Wynsberghe has stressed the importance of the practice-oriented nature of care activities by insisting that the fundamental care values coincide with the moral elements of Tronto’s phases of a care practice. These elements are: attentiveness, responsibility, competence and reciprocity. They form the backbone of a care practice; a way in which one can evaluate good care from bad care. From this perspective good care is achieved if a care activity is fulfilled in an attentive manner, with competence, by a moral agent capable of taking responsibility, while allowing for responsiveness on the part of the care receiver.^5^ Moreover, even if she does not deploy the terminology of coarse- and fine-grained descriptions of actions, van Wynsberghe (2012, 2013, 2015) also recurs to a fine-grained description of a care activity to reveal the many significant internal points of it. Each of the moral elements is defined uniquely for every different practice and is dependent on the context of care and the individual patient (Tronto 1993; van Wynsberghe 2012, 2013).

As this all sounds like an accurate description of what many would consider a good healthcare practice, should we then conclude that: if a care robot would take away from the skills developed, or the fundamental care values that come into being through the practice, then the use of such care robots should always be prohibited or discouraged? Not so fast. What the nature-of-activities approach also allows us to do is to understand that care activities (or at least some portions or aspects of them) may *also* be considered as goal-directed, and this can make the normative evaluation of care robots more complex than allowed by some care ethicists.

## Lifting Under Different Descriptions

In order to better understand the interplay between goal-directed versus practice oriented aspects of care activities, let us look, as a first example, to the activity of lifting a patient. If one were to consider the activity of lifting exclusively in terms of its immediate external goals (as goal-directed), the activity could be described as moving the patient from the bed to the wheelchair in order to bring them to the toilet (or to an appointment etc.). From this perspective the activity of lifting simply consists of: safely raising the patient out of bed at a certain angle, with a certain speed and force, and safely placing them in their wheelchair.

Alternatively, seen through the practice-oriented lens, the same activity appears much more complex. During lifting the patient is vulnerable and responsive, he/she must learn to trust their care giver, and the care giver must establish themselves as an agent to trust (among other things). Lifting is a moment in which the care giver and care receiver form the therapeutic relationship together. This relationship has a value in itself but is also necessary for the later care of the patient; in order for the care receiver to be honest about their symptoms, to take their medication and to comply with their care plan. Lifting is also a moment in which the care giver is able: to assess the neurological and physiological status of the patient; to make eye contact with the patient; and to socially interact with the patient. Thus, *under this practice*-*oriented description*, in order to be properly carried out the activity of lifting requires not only that the care giver efficiently and safely move the patient from one place to another but also that they develop observational skills and meet important general social and medical needs of the patient.

Should the operation of lifting be delegated to robots? In relation to this example, the nature-of-activities approach can be seen as offering two different kinds of contributions to the ethical analysis of care robots. Firstly, it helps make sense of the normative disagreement about the use of robots in care activities. By normative disagreement we refer here to the tension between different normative conclusions that one may draw depending on their conception of the care activity. Once we realize that a given care activity can be *legitimately* described appropriately in different ways, we may also make sense of the presence of a wider range of different, potentially contrasting values embedded in the activity. Seen simply as a process of transport, lifting is an activity that requires the *safest* and most *efficient* means to be fulfilled. Seen as a moment of socialization, trust-building, and care-taking, lifting is a “practice” (in the sense of care ethics) that requires *human responsiveness* and *human* *attentiveness* to be fulfilled. Depending on our preferred *conception* of care, we may have contrasting ethical views about how care should look. In this perspective our conceptual analysis of the care activity is an important tool toward an ethics of care robots, insofar as a detailed and comprehensive account of the values at stake in healthcare and, relatedly, of the different available conceptions of care are held to be an important part of this enterprise.

Moreover, our reconstruction of the normative disagreement about care activities can also help make sense of the value of moral autonomy of patients/users in deciding how to live their lives, as it has been recently stressed by Sorell and Draper (2014). In her (2014) paper Sharkey points out that robots may both enhance and diminish the dignity of elderly persons; they may enhance dignity as they refrain from typically human ways of disrespecting elderly persons, i.e. rude or otherwise offensive behaviour deriving from tiredness, stress, overwork etc.; however, the elderly person’s dignity may also be endangered by robots insofar as current robots cannot give the “real compassion and empathy or understanding” typical of human companionship. Sharkey then points out that “if older people were to be *predominantly* looked after by robots, and as a consequence were not able to have access to human companionship, *many people* would consider their lives to be unacceptably impoverished” (65, emphasis added). However, in the second part of her paper, Sharkey also offers a more nuanced position by distinguishing the impact on dignity of different kinds of robots (assistive, monitoring, companion).

Whereas we agree with Sharkey that we need to give nuanced answers, we also think that the capability approach she proposes offers a not broad enough methodology to make sense of the many relevant values pursued by particular care activities. Our nature-of-activities approach is arguably able to offer a broader perspective. In fact, in relation to particular care activities (e.g. lifting), our approach allows one to recognize the legitimacy of having different understandings of what those activities *are*. For some people in some circumstances lifting *is* just about moving from one place to another; for those people human company or even the presence of other people is not a part of the activity of lifting. Therefore, failing to be able to lift themselves, these patients may just prefer to have a machine rather than a human person supporting them. In other words, an elderly person who endorses this view of the nature of lifting may reasonably prefer to be *enabled* by a machine to safely, efficiently, autonomously lift as opposed to be caringly, compassionately, empathetically *assisted* by a human carer in lifting. For these people care is good enough insofar as it enables them to pursue their goals—that is respecting their moral autonomy—no matter whether pursuing these goals may contribute to the development of a relevant capability or to the promotion of the person’s dignity.^6^

On the other hand, if an elderly person shares the care ethics view that in their condition of physical frailty lifting *is* an important moment of empathetic interaction with a carer moved by compassion, attentiveness, and responsiveness; then they may reasonably refuse to be supported by a robot in this activity, even if the robots were able to guarantee the same or a even higher level of efficiency and safety in the operation of lifting.

What we are here suggesting is simply that when different descriptions of an activity are equally legitimate—as they arguably are in this case—then normative disagreement about the way in which a given activity has to be fulfilled—for instance, the disagreement between an elderly person and her care takers about the way in which she has to be assisted—may also be irreducible, and therefore we would rather leave it to the elderly persons themselves to decide how to be treated. It is a virtue of the nature-of-activities approach that it can make sense of the irreducibility of this conceptual and normative disagreement, and of the related necessity of respecting the patient’s autonomy of choice about their treatment in the presence of this tension between contrasting values.

Certainly, what the limits of autonomy for (mentally competent) patients or users should be is open to debate. Firstly, whereas we may easily accept the idea that in a home setting elderly persons should be able to make their own view of what taking good care of them means, things are more complex in a nursing home setting. In this context there may be stricter, objective limits to the way in which elderly persons may request to be taken care of. In complex institutions like nursing homes, where different actors with different roles, tasks and responsibilities, closely interact with patients on the basis of complex rules and procedures, patients’ preferences simply cannot and should not always be complied with^7^; arguably, in these complex social contexts the assessment of what should count as (good) care cannot and should not left only to the judgements of individual patients. Secondly, according to an example by Sharkey and Sharkey (2012) no matter in what setting they operate, we may not want lifting robots to be allowed to release patients over the side of a high balcony in an apartment building, even if explicitly requested to do so by a mentally competent elderly person. However, such limitations would also apply to the behaviour of an autonomy-respecting human helper (Sorell and Draper 2014). With or without robots, autonomy in healthcare may be in tension with other paramount human values: life and health. As this is a well-known crux in other general debates in medical ethics—typically but not exclusively: assisted suicide, abortion—this is not the place for us to take a position about this broader issue.

A related point that is more specifically relevant to care robots should be mentioned though. The legitimacy of different views on the nature of care activities is arguably a fact that points in favour of respecting all patients’ and users’ autonomous choices in relation to their assistance and care. However, in the interest of making sure that *everyone* is in the position to realize their autonomous choices, we have to make sure that economic and social conditions do not make certain options *de facto* unavailable. From this perspective, given the economic pressure to replace human work with machines, during the process of introduction of robots in care practices we have to create also the social, political, economic conditions for those who do *not* want to be assisted by machines to be in the position of doing so. In the absence of the relevant political, social, economic constraints,^8^ we may run the risk that given the high economic interests at stake, once assistive robots are massively introduced in care institutions and practices, they will be used no matter what the different preferences, values, and needs of patients and users are.

## Urine Sample Collection Under a Fine-Grained Description

In the lifting case the nature-of-activities approach allows us to make sense of the normative disagreement about the use of care robots as well as of the problem of defining the legitimate limits of users’ autonomy; however, in other cases the nature-of-activities approach may support more clear-cut normative conclusions.

As a second example of a healthcare activity, let us consider the urine sample collection in pediatric oncology (van Wynsberghe 2013). A sample of urine must be collected for testing the presence (or lack thereof) of chemotherapy toxins for pediatric patients undergoing chemotherapy. Following the care ethics tradition, we may describe the activity of urine collection as practice-oriented in the sense, for instance, that the activity realizes the care skills of the nurse: the nurse remains attentive to, responsible for, and competent as care provider throughout the activity. Moreover, even the urine sample collection can be seen as a moment in which patients have the chance to get in touch and to briefly interact with a human care-taker. However, it is also true that the collection of the sample has a clear external goal, namely testing the presence (or lack thereof) of chemotherapy toxins for patients.

Now, unlike what happens with the activity of lifting, here the different aspects of the activities can be not only conceptually distinguished but also *materially separated*. When we disentangle what the nurse is actually doing—walking into the patient’s bathroom and taking a sample of urine—it is possible to suggest that the portion of the activity consisting of the sample collection itself is not necessary to ensure attentiveness, responsibility, or competence of the nurse. In other words, sample collection is not a necessary feature to establish attentiveness on the part of the nurse. Accordingly, we may describe at least this portion of the activity as prominently, if not exclusively, goal-directed—its main (and possibly only) goal is to allow for a urine test. Moreover, it has to be noted that the sample collection may also be not only embarrassing for the patient but also dangerous for nurses’ health. Nurses often do not have the time to wear the protective clothing that guards them from the chemotaxins which are able to cross the skin barrier. As a result, nurses put themselves at risk in order to satisfy the goal of sample collection while ensuring the wellbeing of the patient (van Wynsberghe 2013). Is it possible to suggest that a robot could fulfill this segment of the activity?

To be clear, the activity of sample collection is a complex one: its immediate goal is external (to allow for the urine test), but some aspects of the activity are also connected to the patient’s overall care (i.e. are internal). In addition, some portions of the activity are harmful for the nurses’ health. This analysis allows us to formulate the ethical question of the permissibility of the use of care robots for urine sample collection in a more precise way: We should not simply ask whether it would be permissible to remove the nurse *from the activity of sample collection* and to replace her with a robot; rather, we should ask whether it would be permissible to remove the nurse *from a portion* of the activity of sample collection, namely the goal-directed and harmful to the nurse portion; provided that a connection between the nurse and the activity of sample collection is maintained. Put in these terms, the question as to the opportunity of a task delegation to a robot may receive a positive answer.

What we have done up to this point of the paper is to use some tools from the philosophy of action (practice oriented vs. goal-directed activities, different descriptions of actions, and coarse- vs. fine-grained descriptions of activities) to provide a way in which care activities and the values they foster or embed can be articulated and defined with the greatest amount of detail possible. This has allowed us to show the many dimensions of a care activity as well as how a care activity may be disentangled to reveal both external and internal values. We have indicated two ways in which this analysis may be relevant for the ethical evaluation of care robots.

With reference to the example of lifting we have shown how the nature-of-activities approach may help make sense of the legitimate plurality of *perspectives* on what care activities are and should be like: good care may require contact and attentiveness but for some people under some circumstances it may also just require the enablement or restoring of physical capacities, autonomy and independence. With reference to the example of urine sample collection, we have shown how a more fine-grained description of the different aspects constituting a given care activity may help isolate the portions of that activity which can or even should be delegated to robots. We will now show how fruitful the nature-of-activities approach can be once we shift our attention from the ethical evaluation to the *ethical* *design* of a care robot.

## The Design Perspective

The Care Centered Value Sensitive Design (CCVSD) approach provides a framework for designing future care robots in a way that systematically accounts for a recognition of *care values* throughout the design process of the robot. The CCVSD approach aims at incorporating values into the design of care robots such that through the use of the robot care values come into existence (van Wynsberghe 2012, 2013, 2015). This approach relies, on the one hand, on the embedded values concept (Nissenbaum 1998), according to which information technology is not morally neutral insofar as computer systems or software promote or demote particular moral values and norms. On the other hand, CCVSD also relies on the approach of Value-Sensitive Design (VSD) as originally elaborated in computer ethics (Friedman et al. 2003, van den Hoven 2007). According to VSD moral and social values should work as non-functional requirements for the design of a good technology. In her work developing the CCVSD approach, van Wynsberghe has recognized that the capacity to understand care activities is particularly important when considering how to design a care-sensitive robot. Design refers to what capabilities the robot ought to have to determine its role. Understanding a care activity allows a robot designer to create a robot whose functioning is compatible with, or ideally can promote, the realization of care values. What’s more, understanding the distribution of roles and responsibilities entailed in care activities allows robot designers to prudentially choose the roles and responsibility (or lack thereof) delegated to the robot.

The nature-of-activities approach can arguably help develop the CCVSD approach further. Firstly, it helps give a more explicit and solid *philosophical foundation* to the inquiry into the nature of care activities required for a care-sensitive design. Secondly, the nature-of-activities approach *extends* the CCVSD by helping make sense of the possibility of conceptual and normative disagreement about the nature and value of care activities; and thus by leaving some space also for values traditionally downplayed in the care ethics tradition, like individual autonomy. Thirdly, by offering specific conceptual tools for producing richer and *more fine*-*grained descriptions* of care activities and of the values embedded in the different aspects or portions of the activities, the nature-of-activities approach also suggests a way to try to solve or loosen *by design* the tension between different values at stake in a given activity.

In order to better appreciate the importance of the nature-of-activities approach for a care-sensitive design of care robots, let us go back to the case of urine testing and consider van Wynsberghe’s (2013) example of a urine testing robot, referred to in other work by the same author as the “wee-bot” robot. van Wynsberghe has described a way in which the robot can be delegated the role of sample collection while at the same time limiting its responsibility for the completion of the role. To do this the design suggestions are as follows: the robot is designed in a way that it travels autonomously to reach the patient’s room. Once there, it requires information from the nurse to indicate his/her presence. This design consideration enforces that the nurse be present for urine testing. The robot then enters into the patient’s bathroom autonomously to collect the sample; it collects the sample from the patient’s toilet or waste bin (as opposed to attaching to the patient’s organs), and then proceeds to exit the room. It travels to the nurse waiting outside the bathroom (or the patient’s room) and again confirms the presence of the nurse. The robot transmits the information that it has obtained the sample (and perhaps has also already done the testing). With this information the nurse can choose to send the robot (carrying the sample) to the oncology lab or alternatively can select to have the robot complete the analysis and send the results on to the oncologist. Whatever the nurse decides he/she is aware that the sample collection has happened and that he/she is responsible for passing on the results of the sample collection to the oncologist. This design suggestion is intended to enforce that a human agent is responsible for the successful completion of sample collection.

From a design perspective the nature-of-activities approach may help identify a broad variety of relevant ethical requirements for the design of a care robot, a variety arguably broader than that highlighted by specific approaches based on one specific ethical perspective (care ethics, capabilities approach, autonomy). In the “wee-bot” example just illustrated, our approach has allowed us to identify two distinct sets of goals to be achieved in the activity of urine testing, i.e. the (external) goal of the safe and efficient collection of a sample to be tested and the (internal) goal of the realization of the values of attentiveness and responsibility on the part of the nurse. This has in turn allowed us to identify some design requirements of an ethically acceptable “wee-bot”; the ability to safely collect the urine sample, by removing the nurse from harm, while at the same time: (a) allowing the nurse to remain connected to both the patient and the patient’s care by being present for the activity of sample collection, and (b) preserving the nurse’s accountability for the process.

## Where Does this Bring Us?

The consequences of this discussion apply to the designers and developers of care robots as well as to the ethicists engaged in critical reflections on the design of care robots. In order to answer the question as to *when* *and how care robots should be used* and *how care robots should be designed* we need first to reflect on what the different points of care activities are, and how these different points are realized in different versions, aspects or portions of those activities. We have shown here some preliminary thoughts on the relevance of the nature-of-activities approach for the ethics of care robots by discussing the example of lifting and urine testing; we have then presented the case of the hypothetical “wee-bot” robot to illustrate the fruitfulness of this approach from the perspective of Care Centered Value Sensitive Design (CCVSD).

Future research could benefit from engaging in a systematic documentation of different values embedded in care activities according to a distinction between goal-directed and practice-oriented (aspects of) activities, and according to different legitimate (fine-grained) descriptions of said activities. This requires a combination of: (a) philosophical reflection and conceptual analysis; b) deep understanding of the dynamics of the hospital and/or nursing home and the roles and responsibilities of care givers, (c) a standing attention to the way in which care activities and care technologies are perceived and evaluated by patients and users.^9^

## Footnotes

1 This is the *use* perspective; for the *design* perspective, see “The Design Perspective” section below.

2 Their distinction is in turn inspired by Rawls (1955), McIntyre (1985) and Searle (1995).

3 Apart from them creating unjustified risks for athletes’ health and safety.

4 See “Introduction” section above for an explanation of our reasons for using the term “care activity” instead of “care practice”.

5 A somehow similar approach to what he calls “good care” has been endorsed by Coeckelbergh (2010).

6 In their (2014) Draper et al. present the interesting case of elderly people who want the robots helping them to pursue morally dubious activities like gambling.

7 This may also require different technological solutions; for instance, whereas it would be possible to imagine a remote control in the hands of a patient or elderly person using a lifting robot in a home setting, this would not be recommendable in a nursing home setting.

8 Some of these constrains should be taken into account also in the *design* of robots, see “The Design Perspective” section below.

9 Some steps in the direction of mapping the perception of different potential stakeholders involved in the use of care robots have been recently taken by Draper et al. (2014).
